# The “Let’s Talk about Children” intervention in a Finnish school context: fidelity, parents’ experiences, and perceived benefits

**DOI:** 10.3389/fpsyg.2023.1183704

**Published:** 2023-06-09

**Authors:** Lotta Allemand, Mika Niemelä, Marko Merikukka, Katariina Salmela-Aro

**Affiliations:** ^1^Faculty of Educational Sciences, University of Helsinki, Helsinki, Finland; ^2^Faculty of Medicine, Research Unit of Population Health, University of Oulu, Oulu, Finland; ^3^Itla Children’s Foundation, Helsinki, Finland; ^4^Research Service Unit, Oulu University Hospital, Oulu, Finland

**Keywords:** Let’s Talk about Children, school intervention, children’s well-being, resilience, mental health promotion, fidelity, parent–teacher collaboration, parenting

## Abstract

The Let’s Talk about Children intervention is a tool for parents and professionals to work together to promote children’s positive development, resilience, and psychosocial well-being in social and healthcare services, at school, and in day care. The aim of this study was to evaluate the fidelity, parents’ experiences, and perceived benefits of using the Let’s Talk about Children intervention in a school context. Participants (*N* = 65 first-grader parents) completed an online questionnaire after the intervention. The results show that the intervention was delivered as designed and conducted with high fidelity. Parents’ experiences of the Let’s Talk about Children discussions were positive, parents felt that the atmosphere was good during the discussion, and the participants reported benefits from the intervention.

**Clinical trial registration:**ClinicalTrials.gov, identifier NCT05038280.

## 1. Introduction

There is a growing concern about the mental health and psychosocial well-being of children and adolescents globally (United Nations Children’s Fund, 2021). In Finland, 10–15% of children and 20–25% of adolescents have mental health problems (Aalto-Setälä et al., 2020). Thus, protecting and promoting children’s and adolescents’ mental health, psychosocial well-being, and overall development at school is crucial (Thompson et al., 2020; World Health Organization, 2021). One option to do this is to implement school-based preventive and promotive programs that aim to enhance school children’s and adolescents’ mental health and psychosocial well-being (e.g., Bywater and Sharples, 2012; Dray et al., 2017; O’Reilly et al., 2018). One of these programs is the Let’s Talk about Children intervention (LTC intervention), which is a tool for parents and professionals to work together to promote children’s positive development, resilience, and psychosocial well-being in social and healthcare services, at school, and in day care (Solantaus et al., 2009; Niemelä et al., 2019).

The LTC method is one of the Effective Child and Family Methods developed as a part of the Effective Child & Family project coordinated by the Finnish Institute for Health and Welfare. The LTC intervention is distributed through the non-governmental organization MIELI Mental Health Finland (n.d.). The LTC intervention was originally developed for adult health services to support parents and children with parental mental health problems (Solantaus and Toikka, 2006; Solantaus et al., 2009). From the beginning, the LTC has been used widely in varying municipal social and healthcare services, but also in schools and day care (Niemelä et al., 2019). The LTC intervention is also used as a part of a comprehensive collaboration model to enhance children’s psychosocial well-being and service integration (Niemelä et al., 2019; Takalo et al., 2022).

The LTC method is based on an ecological, transactional model of child development (e.g., Bronfenbrenner and Ceci, 1994; Sameroff, 2010; Ungar et al., 2013; Solantaus and Niemelä, 2016). The child, family, and other developmental contexts like day care, school, and leisure time are part of the ecology, and a child’s interaction with the environment is essential for a child’s development and resilience (Bronfenbrenner and Ceci, 1994; Sameroff, 2010; Ungar et al., 2013). The LTC work is based on the idea that a child’s favorable development, well-being, and mental health will develop in interaction with the child’s developmental contexts (Solantaus and Niemelä, 2016; Wahlbeck et al., 2017). Thus, the everyday interaction between the child and their family, school, and leisure activities is crucial (Solantaus, 2002). The action and interaction at home and school can lead the child’s development in a favorable or unfavorable direction (Solantaus and Niemelä, 2016), and support in other developmental contexts is essential when there are problems in others (Solantaus, 2002; Wahlbeck et al., 2017). In addition, the LTC work emphasizes the interaction between different developmental contexts, e.g., home and school, in promoting a child’s positive development and psychosocial well-being (Niemelä et al., 2019).

In this study, we examine the LTC intervention in a school context. The school system in Finland consists of 9 years of basic education: 6 years of primary school (grades 1–6) and 3 years of lower secondary education (grades 7–9). Children start primary school in the year they turn seven. In 2021 reform, compulsory education was extended from 16 to 18  years. Thus, adolescents are required to continue their studies after 9th grade in general upper secondary education or vocational education and training. Most schools in Finland are public, and basic education is free for all students. Schools follow the national core curriculum, but the municipalities organize education. However, the responsibility to organize school healthcare and welfare services was transferred from municipalities to the well-being services counties at the beginning of 2023. The student welfare services focus on preventive work to support students and the whole school community’s well-being. In addition, students are entitled to individual welfare services (e.g., school nurses, school doctors, school psychologists, and curators). The implementation and the use of LTC are decided by the local education authorities, and schools decide how the LTC method is implemented.

The LTC method has been developed and adapted to respond to the context in which it has been implemented (Allchin and Solantaus, 2022). The school version of the LTC intervention was developed in collaboration with mental health professionals, schools, teachers, and families. The aims and practical implementations of the LTC method at school are described in the logbooks (Solantaus, 2021). The logbooks are published in many languages (e.g., Finnish, Swedish, Russian, and English) and can be found at the MIELI Mental Health Finland website.^1^ Two age-appropriate logbooks are used in a school environment, one for primary school (for children aged 7–12) and the other for lower secondary education (for children aged 13–15). The LTC intervention includes the LTC discussion (1–2 sessions per family) and the network meeting. The LTC network meeting will be organized after the LTC discussion(s) if there is a need for cross-sectoral collaboration with the services outside school to promote a child’s well-being. In schools, the LTC intervention can be used universally for all children or as a selective or targeted intervention for children at risk of facing or already having problems. The universal approach suggests that the intervention aims to prevent problems and improve all children’s well-being in the school/class. When the LTC intervention is used universally at school, it is offered to all students in specific grades and their parents/other caregivers. In a school environment, LTC discussions are held with teachers. Before using the LTC method in their work, teachers receive intervention training. The training is two full days (12 h) and fieldwork at least with two families. Supervision and consultation on how to carry out the intervention are provided during the training.

The LTC intervention is an opportunity for the children’s parents and teachers to discuss and share thoughts about the child’s everyday life and well-being, build a shared understanding of the child’s situation, and plan activities based on this shared understanding. The LTC intervention is also a way for parents and teachers to get to know each other and build a collaborative relationship based on mutual support. In addition, the LTC intervention aims to support teachers in their work and parents in their activities with the child. The LTC discussion highlights reciprocity, and parents/other caregivers and teachers both bring their expertise to the discussion, with parents/other caregivers being experts concerning the child’s everyday life and the family situation and teachers being professionals in learning and teaching. In the LTC discussion, the parents/other caregivers, the teacher, and the child discuss the child’s everyday life in all developmental contexts at school, at home, and in leisure time. The participants discuss, e.g., the child’s well-being, mood and energy, the child’s curiosity and joy of learning, the child’s sense of belonging to the school and the school engagement, the child’s school achievement, the child’s friend situation and leisure time activities and social situations, interactions, disagreements, and conflicts at home, at school, and with friends. As part of the discussion, teachers and parents/other caregivers also plan how both of them could enhance the child’s identified strengths and give support in vulnerabilities at home and school. In the LTC model, strengths mean social situations which go well in children’s everyday life, not any specific skills or talents the child may have. Vulnerability, on the other hand, is something that is already a problem or might become a problem in a child’s life.

The previous studies of the LTC intervention have focused on the implementation and sustainability of the method and the intervention outcomes for parents and children in adult psychiatry and adult mental health services (Allchin and Solantaus, 2022). However, studies have also been conducted in other areas, such as parental cancer (e.g., Niemelä et al., 2010, 2012), gambling (e.g., von Doussa et al., 2017), and community studies (e.g., Niemelä et al., 2019; Takalo et al., 2022). The previous LTC studies were carried out on a range of populations in Finland (e.g., Solantaus et al., 2009, 2010; Niemelä et al., 2012; Punamäki et al., 2013), Australia (e.g., Maybery et al., 2019; Goodyear et al., 2022), Japan (e.g., Ueno et al., 2019) and Greece (e.g., Giannakopoulos et al., 2021). The LTC studies vary extensively in research methodology, some being randomized controlled trials (e.g., Punamäki et al., 2013; Giannakopoulos et al., 2021), some being qualitative (e.g., von Doussa et al., 2017) or mixed method studies (Maybery et al., 2019) and some studies being descriptive in nature (e.g., Niemelä et al., 2016).

The previous research results have been promising. As Allchin and Solantaus (2022) presented in their article, the LTC intervention has been shown to be effective and acceptable in a range of settings with varied populations. The high fidelity and feasibility of the LTC intervention have been demonstrated in previous clinical trials in healthcare services (Solantaus et al., 2009; Ueno et al., 2019). Moreover, parents with mental health problems have reported intervention to be beneficial relating to their self-reported well-being and parenting, and parents have also experienced the intervention to be helpful or useful (Solantaus et al., 2009; Ueno et al., 2019). A recent randomized controlled trial study in Greece revealed that the LTC intervention reduced depressed parents’ self-reported symptoms of depression and anxiety and improved perceived social support, parenting, and family functioning (Giannakopoulos et al., 2021). The same study also showed that the LTC intervention reduced depressed parents’ children’s emotional/behavioral problems and improved children’s well-being, prosocial behavior, and health-related quality of life (Giannakopoulos et al., 2021). However, it should be noted that there was no passive control group in this study (Giannakopoulos et al., 2021). The LTC intervention has also been used in treating parents with cancer to support their parenting, and a study indicated that the intervention reduced cancer patients and their spouses’ psychiatric symptoms (Niemelä et al., 2012). In addition, in one community-level study, it was found that using the comprehensive Let’s Talk about Children model leads to a reduction in the community’s referrals to child protection services (Niemelä et al., 2019). However, the previous research on LTC intervention in a school environment is limited, and to fill this research gap, we are investigating LTC intervention in a school context.

This study is a part of the ongoing Let’s Talk about Children in a School Context intervention study to be conducted in 2021–2025. The first aim is to examine LTC intervention fidelity in a school context. Intervention fidelity is acknowledged as an important area requiring further attention (Carroll et al., 2007; Gearing et al., 2011), but only a few intervention studies have reported on fidelity (Smith et al., 2007; Maynard et al., 2013). However, there seems to be an increased interest in studying fidelity and linking fidelity to intervention outcomes in a school environment (Rojas-Andrade and Bahamondes, 2019). Previous research indicates that intervention fidelity is associated with better intervention outcomes (Durlak and DuPre, 2008); notably, two components of fidelity, students’ exposure and receptiveness to the intervention, are shown to be associated with the outcomes of school-based mental health interventions (Rojas-Andrade and Bahamondes, 2019). In this study, we replicate the previous LTC study made in healthcare services (Ueno et al., 2019), and intervention fidelity is defined as the degree to which intervention is put into practice as designed (Dusenbury et al., 2003; Carroll et al., 2007). The high fidelity of the LTC intervention has been demonstrated in previous clinical trials in healthcare services (Solantaus et al., 2009; Ueno et al., 2019), and we expect that the intervention will also be conducted with high fidelity in a school environment (hypothesis 1).

The second aim of this study is to examine, report, and identify the areas of the parents’ experiences and perceived benefits of using the LTC intervention in a school context. Starting primary school is an important transition in children’s lives, and studies have shown that a good relationship between parents and teachers is one of the key factors for a successful transition when children start school (Peters, 2010). The LTC discussion aims to build a collaborative relationship between parents and teachers, and it is vital to know whether the LTC discussion is an appropriate tool for this. It is also acknowledged that related to the new interventions; it is important to know how individuals perceive the advantages of new interventions, not only how advantageous the intervention is objectively (Rogers, 2002). Previous LTC studies have shown that parents have experienced the intervention as being helpful or useful (Solantaus et al., 2009; Ueno et al., 2019) and beneficial relating to their parenting in adult mental health services (Solantaus et al., 2009; Ueno et al., 2019; Giannakopoulos et al., 2021). Based on this, we expect that the parents perceive the intervention to be useful and beneficial in a school context as well (hypothesis 2).

## 2. Materials and methods

### 2.1. Participants and procedure

In most schools where the LTC intervention is implemented, it is used universally and offered to all first-grade students and their parents/other caregivers. Thus, in this study, we examined the first-grade students’ parents. The data used in the present study were collected in 2021 during the autumn semester. A total of 76 schools in four municipalities in Finland were invited to participate in the study. Approvals for this study were initially obtained from the directors of education of all the municipalities. The data were collected in real-life settings in schools in which the LTC intervention is used universally, is already implemented, and the teachers use the LTC intervention in their work. The schools were invited to participate in the study in an email sent to school principals containing information about the research. Nine schools registered for the study, but four different schools (five school classes) in four municipalities eventually participated. To calculate the response rate of this study, it was estimated that every class that participated in the study contained 20 students. In Finland, the average group size is 19.6 students in primary schools (OECD, 2017). Teachers provided parents with an opportunity to fill out the online questionnaire immediately after the LTC discussion. The parents responded to the questionnaire either alone or together; thus, there was one response from each family. Sixty-five out of the 100 parents responded to the questionnaire, meaning the response rate in this study was 65%. Participation in the study was voluntary, and before answering the questions, parents were asked to provide their informed consent. The questionnaire took about 10 min to complete. The study protocol was approved by the University of Helsinki Ethics Review Board in the Humanities and Social and Behavioral Sciences. The trial has been registered in the ClinicalTrials.gov registry (NCT05038280).

### 2.2. Measures

#### 2.2.1. Demographic characteristics

Demographic information collected from parents in the questionnaire included mother/father/other caregivers, age, size of hometown, marital status, birthplace, native language, education level, and professional status. Participants’ demographic characteristics are presented in Table 1. The sample consisted of 65 parents (70.8% mothers, 29.2% fathers). The mean age of participants was 37.7 years (SD = 5.1 years). Nearly 97% of the participants were born in Finland and spoke Finnish as their native language, 76.9% of the participants were married/in registered partnerships/or cohabiting, and 53.8% of participants lived in a mid-sized or a small city while 40.0% of the participants lived in a large city. Of the participants, 72.3% had a bachelor’s or master’s degree, and 78.5% were employed.

**Table 1:** Demographic characteristics.

	*n*	%
*Parents*		
Mother	46	70.8
Father	19	29.2
Other caregivers	0	0.0
*Marital status*		
Single	6	9.2
Married or registered partnership	34	52.3
Cohabitation	16	24.6
Divorced	9	13.8
*Birthplace*		
Finland	63	96.9
Other	2	3.1
*Native language*		
Finnish	63	96.9
Other	1	1.5
*Location of residence*		
Large city (more than 100,000 inhabitants)	26	40.0
Mid-sized or small city (under 100,000 inhabitants)	35	53.8
Population center in a rural area	1	1.5
Sparsely populated rural area	3	4.6
*Professional training*		
Basic education (comprehensive school)	1	1.5
General upper secondary education	1	1.5
Vocational education and training	16	24.6
Bachelor’s degree	28	43.1
Master’s degree	19	29.2
*Labor market situation*		
Employed	51	78.5
Full-time student	3	4.6
Unemployed/laid-off	3	4.6
Stay-at-home mother/father	4	6.2
Other	4	6.2

*N* = 65. Participants were an average 37.7 years old (SD = 5.1 years, Median = 38.0, Q1-Q3 33.50–40.00).

#### 2.2.2. Fidelity

Fidelity was measured by intervention delivery. The LTC discussions are carried out with fidelity if the children’s everyday life at school, at home, and in leisure time, and the strengths and vulnerabilities concerning everyday life were discussed following the developmental log used in the LTC discussions (Ueno et al., 2019). We obtained parents’ feedback to assess fidelity using a self-report questionnaire. Originally this questionnaire was developed to assess the fidelity of the LTC intervention in adult mental health services in Japan (Ueno et al., 2019). The scale was modified to fit better in a school environment. The scale consisted of nine items (e.g., “Did you discuss the child’s everyday life at home/school/in leisure time during the LTC discussion,” “Did you discuss the child’s strengths/vulnerabilities during the LTC discussion”). The participants were asked to choose between two alternatives “yes” or “no.” The high percentage of positive responses (“yes”) was regarded as an indicator of the high fidelity of the intervention. In addition, because one aim of the LTC discussion is to build a shared understanding of the child’s situation and identify the child’s strengths and vulnerabilities, we asked parents if the discussion made them see some strengths/vulnerabilities in their child that they have not thought of earlier or if the discussion made parents to identified only those strengths/vulnerabilities in their child that they had already thought of or if the discussion made parents to the see that their child had less strengths/vulnerabilities than they had thought of. In this section, there were three alternatives, and the participants were asked to choose the choice they agreed with.

#### 2.2.3. Parents’ experiences and perceived benefits of the intervention

The parents’ experiences and perceived benefits of the intervention were measured through the questionnaire developed for this study. We based the questionnaire on the original questionnaire, which assessed the perceived benefits and families’ experiences of the LTC intervention in adult mental healthcare services in Finland and Japan (Solantaus et al., 2009; Ueno et al., 2019). The original questionnaire consisted of 22 items measuring families’ experiences, parents’ self-understanding, mutual family understanding, parenting, future orientation, well-being, treatment motivation, and child-related worries (Solantaus et al., 2009; Ueno et al., 2019). Eight items were included from the original scale to the new scale. These eight items were selected based on their suitability for the school environment. We further modified the items to fit the school context better. The omitted items were related to health services or parental mental health problems (e.g., the importance of the intervention to the parent’s own treatment and if the intervention had impacted on parent’s self-acceptance or guilt) and thus did not fit the school context. The original scale rating (e.g., “very positive change,” “positive change,” “no change,” “negative change,” “very negative change”) was changed to a 5-point Likert scale (1 = totally disagree, 2 = somewhat disagree, 3 = neither agree nor disagree, 4 = somewhat agree, 5 = totally agree). In addition, four new items were formulated and included in the scale to evaluate the usefulness of the intervention and the atmosphere of the discussion (e.g., Vacco, 2002). The new scale consisted of 12 items (e.g., “I think that the discussion was useful”) measuring the parents’ experiences and perceived benefits. The Cronbach’s alpha for all 12 items was 0.83. All 12 items are presented in Table 2.

**Table 2:** Items measuring the parents’ experiences and perceived benefits of the LTC intervention in a school context (12 items).

1. I think that the discussion was useful. (original)
2. I think that the LTC discussion is a good method for increasing teacher–parent collaboration. (new)
3. I had a positive reaction to this discussion. (new)
4. The discussion increased my understanding of my child’s everyday life at school. (original)
5. The discussion increased my confidence in my child’s future. (original)
6. The sense of adequacy as a parent increased because of the discussion. (original)
7. Confidence in parenting increased because of the discussion. (original)
8. I got new ideas or tools for my own parenting. (original)
9. The atmosphere was good during the discussion. (new)
10. I felt like I got to say all the things I wanted to say. (original)
11. I experienced discomfort during the discussion. (new)
12. My worries about my child decreased because of the discussion. (original)

Original = items from the original questionnaire (Solantaus et al., 2009; Ueno et al., 2019), new = new formulated items (e.g., Vacco, 2002).

### 2.3. Statistical methods

A principal component analysis (PCA; Jolliffe, 2002) was conducted to identify the areas of the parents’ experiences and perceived benefits. Prior to the analysis, the Kaiser-Meyer-Olkin Test (KMO) and Bartlett’s Test of Sphericity were performed to test the suitability of the data for the PCA. The Kolmogorov–Smirnov test of normality was conducted to test the normality of the data. The internal consistency was measured using Cronbach’s alpha. The means, standard deviations, and Spearman’s correlations of all the examined components were calculated. The data was analyzed using SPSS 28.0 (IBM Corp, 2021).

## 3. Results

The frequency distributions of responses to the statements of the fidelity of the LTC intervention are presented in Table 3. Regarding parents’ feedback, the LTC intervention was conducted with high fidelity. Nearly all parents answered that they discussed their child’s everyday life at school (98.5% of the parents.), at home (98.5% of the parents), and in leisure time (95.4% of the parents) and the strengths (93.8% of the parents) and vulnerabilities (92.3% of the parents) concerning their child’s everyday life in the discussions. In addition, 84.6% of the parents answered that they discussed how their child could be supported in the vulnerabilities by his/her teacher, and 76.9% answered that they also discussed during the discussion how the parents themselves could support their child in their vulnerabilities. Moreover, 83.1% of the parents answered that they discussed how they could enhance their child’s strengths, and 87.7% answered that they discussed how their child’s teacher could enhance their child’s strengths at school. Concerning the child’s strengths, 63.1% of the parents answered that the discussion identified only those strengths in their child that they had already thought of. While 35.4% answered that the discussion made them see such strengths in their child that they had not thought of earlier. Concerning the vulnerabilities, 72.3% of the parents answered that the discussion identified only those vulnerabilities in their child that they had already thought of, 12.3% answered that the discussion made them see such vulnerabilities in their child that they had not thought of earlier, and 12.3% answered that the discussion made them see that their child had less vulnerabilities that they had thought of.

**Table 3:** The frequency distributions of responses to the statements of the fidelity of the LTC intervention.

	% (*N*)
Did you discuss the child’s everyday life at home during the LTC discussion?	
Yes	98.5 (64)
No	1.5 (1)
Did you discuss the child’s everyday life at school during the LTC discussion?	
Yes	98.5 (64)
No	0.0 (0)
Did you discuss the child’s everyday life in leisure time during the LTC discussion?	
Yes	95.4 (62)
No	3.1 (2)
Did you discuss the child’s strengths during the LTC discussion?	
Yes	93.8 (61)
No	6.2 (4)
Did you discuss how the strengths could be enhanced by parents?	
Yes	83.1 (54)
No	15.4 (10)
Did you discuss how the strengths could be enhanced by teacher?	
Yes	87.7 (57)
No	12.3 (8)
Concerning the strengths, tick the choice you agree with:	
1. The discussion made me see such strengths in my child that I had not thought of earlier.	35.4 (23)
2. The discussion identified only those strengths in my child that I had already thought of.	63.1 (41)
3. The discussion made me see that my child had less strengths than I had thought of.	0.0 (0)
Did you discuss the child’s vulnerabilities during the LTC discussion?	
Yes	92.3 (60)
No	7.7 (5)
Did you discuss how the child could be supported in the vulnerabilities by parents?	
Yes	76.9 (50)
No	20.0 (13)
Did you discuss how the child could be supported in the vulnerabilities by teacher?	
Yes	84.6 (55)
No	15.4 (10)
Concerning the vulnerabilities, tick the choice you agree with:	
1. The discussion made me see such vulnerabilities in my child that I had not thought of earlier.	12.3 (8)
2. The discussion identified only those vulnerabilities in my child that I had already thought of.	72.3 (47)
3. The discussion made me see that my child had less vulnerabilities than I had thought of	12.3 (8)

PCA (Promax with Kaiser Normalization) was conducted to identify the areas of the parents’ experiences and perceived benefits. Prior to the analysis, the criteria for PCA were evaluated using the KMO and the Bartlett’s Test of Sphericity. The data are suitable for PCA if the KMO value is higher than 0.60 (Kaiser, 1974) and if the Bartlett’s Test of Sphericity is statistically significant (value of *p* < 0.05). The KMO measure was 0.77, and the Bartlett’s test of sphericity was significant (*χ*^2^ (45) = 369.45, *p* < 0.00), supporting the data’s suitability for PCA. The data were analyzed with both varimax and promax rotation techniques, and we determined that the Promax with Kaiser normalization was most appropriate because of the clear loadings and easier interpretation of the results. During the analysis, two items (“I experienced discomfort during the discussion” and “My worries about my child decreased because of the discussion”) were excluded because they failed to load highly on one of the components or they loaded equally on more than one component. In addition, if these two items were included in the components, they would have decreased the components’ Cronbach’s alphas significantly. Kaiser’s eigenvalue-greater-than-one criteria, the scree plot, and the interpretability of the solution suggested three components. Three components with Eigen values greater than one explained 75.51% of the variance. Eigenvalues, percentages of variance, and cumulative percentages for components for 10 items are presented in Table 4. The first component explained 46.92% of the variance, had loadings from five items (e.g., “I think that the discussion was useful”), and was labelled “experiences and benefits.” The second component explained 17.24% of the variance, had loadings from three items (e.g., “Confidence in parenting increased because of the discussion”), and was labelled “parenting benefits.” The third component explained 11.36% of the variance, had loadings from two items (e.g., “The atmosphere was good during the discussion”), and was labelled “atmosphere of the discussion.” Cronbach’s alphas were 0.86 for experiences and benefits, 0.82 for parenting benefits, and 0.82 for the atmosphere of the discussion. The rotated component matrix of the experience and perceived benefits of the LTC intervention is presented in Table 5.

**Table 4:** Eigenvalues, percentages of variance and cumulative percentages for component for 10 items.

Component	Eigenvalue	% of variance	Cumulative %
1 Experiences and benefits	4.69	46.92%	46.92%
2 Parenting benefits	1.72	17.24%	64.16%
3 Atmosphere of the discussion	1.14	11.36%	75.51%

**Table 5:** The rotated component matrix of the experience and perceived benefits of the LTC intervention.

	Component 1	Component 2	Component 3
	Experiences and benefits	Parenting benefits	Atmosphere of the discussion
Items	Loadings		
1. I think that the discussion was useful.	0.84		
2. I think that the LTC discussion is a good method for increasing teacher–parent collaboration.	1.01		−0.38
3. I had a positive reaction to this discussion.	0.68		
4. The discussion increased my understanding of my child’s everyday life at school.	0.80		
5. The discussion increased my confidence in my child’s future.	0.59		
6. The sense of adequacy as a parent increased because of the discussion.		0.93	
7. Confidence in parenting increased because of the discussion.		0.75	
8. I got new ideas or tools for my own parenting.		0.88	
9. The atmosphere was good during the discussion.			0.86
10. I felt like I got to say all the things I wanted to say.			0.96

Extraction method: principal component analysis. Rotation method: Promax with Kaiser normalization.

The Kolmogorov–Smirnov test indicated that the variables do not follow a normal distribution: experience and benefits D (65) = 0.226, *p* < 0.001, parental benefits D (65) = 0.159, *p* < 0.001, and atmosphere of the discussion D (65) = 0.526, *p* < 0.001. The means, standard deviations, and Spearman’s correlations between all the examined components are shown in Table 6. As the means of the components show, parents’ experiences of the LTC discussion were positive, and parents perceived the intervention to be beneficial (4.62), nearly all parents felt that the atmosphere was good during the discussion (4.92), and parents reported benefits from the intervention related to their parenting (3.94). In addition, the percentage frequency distribution of all responses to the items is presented in Table 7. The highest level of agreement (totally agree/somewhat agree) corresponds to the items “I had a positive reaction to this discussion” (98.4% of the parents), “The atmosphere was good during the discussion” (98.4% of the parents), “I felt like I got to say all the things I wanted to say” (98.4% of the parents), “The discussion increased my understanding of my child’s everyday life at school” (95.4% of the parents), “I think that the LTC discussion is a good method for increasing teacher–parent collaboration” (93.8% of the parents) and “I think that the discussion was useful” (92.3% of the parents).

**Table 6:** The means, standard deviations, and Spearman’s correlations between all of the examined components.

	*M*	*SD*	1	2	3
1. Experiences and benefits	4.62	0.51	–		
2. Parenting benefits	3.94	0.79	0.523**	–	
3. Atmosphere of the discussion	4.92	0.30	0.307*	0.166	–

**Correlation is significant at the 0.01 level, *Correlation is significant at the 0.05 level.

**Table 7:** The percentage frequency distribution of all responses.

Items	Totally agree/somewhat agree%	Neither agree nor disagree %	Somewhat disagree/totally disagree%
I think that the discussion was useful.	92.3	4.6	3.1
I think that the LTC discussion is a good method for increasing teacher–parent collaboration.	93.8	6.2	0.0
I had a positive reaction to this discussion.	98.4	1.5	0.0
The discussion increased my understanding of my child’s everyday life at school.	95.4	3.1	1.5
The discussion increased my confidence in my child’s future.	86.1	12.3	1.5
The sense of adequacy as a parent increased because of the discussion.	64.6	30.8	4.6
Confidence in parenting increased because of the discussion.	69.2	27.7	3.0
I got new ideas or tools for my own parenting.	58.5	38.5	3.0
The atmosphere was good during the discussion.	98.4	1.5	0.0
I felt like I got to say all the things I wanted to say.	98.4	1.5	0.0

## 4. Discussion

The LTC intervention is a tool for parents and professionals to work together to promote children’s positive development, resilience, and psychosocial well-being in social and healthcare services, at school, and in day care (Niemelä et al., 2019). The aim of this study was to evaluate the fidelity, parents’ experiences, and perceived benefits of using the LTC intervention in a school context. The results show that the intervention was conducted with high fidelity, parents’ experiences of the LTC discussion were positive, and parents perceived the intervention to be beneficial. Most parents felt that the atmosphere was good during the discussion, and parents reported benefits from the intervention related to their parenting.

Strengthening children’s well-being and overall mental health is crucial, and schools provide a good context for well-being-promoting interventions (Thompson et al., 2020; World Health Organization, 2021). Almost all children attend school in Finland, and school-based interventions can reach the whole child population and children’s families. Thus, also those children and families who are facing adversities such as a child’s mental health problems but are not using any social or mental health services (Lempinen et al., 2019) are met at schools. If widely applied from day care to schools, the intervention affects many children and their parents in Finland. That is why it is essential to research LTC intervention in a school context.

The first aim of the study was to examine LTC intervention fidelity in a new context, school settings. Fidelity was measured by intervention delivery. We obtained participants’ feedback to assess fidelity using a 9-item self-report questionnaire. Regarding parents’ feedback, the intervention was conducted with high fidelity. The research revealed that the teachers and parents did discuss children’s life, strengths, and vulnerabilities at school, at home, and in leisure time following the developmental log that was used in the discussions. This research result is what we expected (hypothesis 1) and is in line with the previous studies in which the LTC interventions have shown high fidelity in other settings (Solantaus et al., 2009; Ueno et al., 2019). Furthermore, previous research has shown that classroom teachers have been successful in conducting universal interventions such as social and emotional learning programs (Durlak et al., 2011) and psychosocial interventions (Franklin et al., 2017) as part of their work. In Finland, teachers are highly educated and have a university degree which might also be due to their competence to deliver different psychosocial interventions with high quality. In addition, before using the intervention, teachers received intervention training, and intervention training is one of the key elements of intervention implementation and fidelity (Dusenbury et al., 2003; Gearing et al., 2011). Teachers also used logbooks that standardized the intervention.

One aim of the LTC discussion is to identify a child’s strengths and vulnerabilities and build a shared understanding between the teacher and the parents/other caregivers of the child’s situation in different developmental contexts (Solantaus, 2021). Thus, as part of the study, we asked parents if the discussion increased their understanding of their child’s everyday life at school and if the discussion made them see only those strengths and vulnerabilities they already thought of, or did the discussion changed how they saw their child’s strengths and vulnerabilities. The results indicate that for most parents, the discussion increased their understanding of their child’s life at school, and at least for some parents, it increased their understanding of their child’s strengths and vulnerabilities. This is an important result because parents being able to support their children in their school life, need to know how their children are doing at school, which strengths to enhance, and which vulnerabilities to support.

The second aim of this study was to examine, report, and identify the areas of the parents’ experiences and perceived benefits of using the LTC intervention in a school context. The LTC intervention target is not only the individual child but also the teachers and parents as part of promoting students’ well-being. That is why it is important to examine how parents perceive the intervention. This research revealed that parents’ experiences of the LTC discussion were positive, parents felt that the atmosphere was good during the discussion, and parents reported benefits from the intervention. The research results are consistent with the previous studies of LTC intervention in other settings (Solantaus et al., 2009; Ueno et al., 2019; Giannakopoulos et al., 2021), and as we assumed, the parents perceive the intervention to be useful and beneficial in a school context as well (hypothesis 2). Most parents thought that the LTC discussion was a good method for increasing teacher–parent collaboration. This result is important because the aim of the LTC intervention is to build a collaborative relationship between parents and teachers based on respect and mutual support. Furthermore, previous studies have shown that a mutual relationship between parents and teachers is critical to a successful transition when children start school (Peters, 2010). However, at the same time, communication between parents and teachers decreases when children move from prior-to-school settings to school settings (Murray et al., 2015). Thus, some form of partnership activity between parents and teachers is much needed. This study showed that the LTC discussion seems to be a good way for parents, teachers, and a child to talk about the child’s life and well-being while building collaboration between teachers and parents.

### 4.1. Strengths and limitation

There are several strengths in this study. First, previous research on LTC intervention in a school context is limited, and this research brings new knowledge about the intervention in school settings. Secondly, the data used in this study were gathered in real-life settings in schools where the LTC intervention had already been implemented, and the teachers used the LTC intervention in their work. As previous LTC research has shown, an “*in-situ*” model is effective at developing evidence-based practices (Allchin and Solantaus, 2022), and for the future development of the intervention, it is essential to get knowledge about the intervention in real-life settings. The third strength of this study is that it made the voice of the parents heard. However, in the future, more research is needed to capture the teachers’ and different-aged students’ experiences of LTC intervention in a school context as well. There are also some limitations in this research. First, there was no control group in this study. Secondly, we used a self-report questionnaire to measure parents’ experiences and perceived benefits of the LTC intervention. A self-report questionnaire only gives responses to items included in the scale and may be limited in capturing all the parents’ thoughts on the intervention. Further research is needed using different research methodologies to get a better understanding of the participants’ thoughts and experiences of the LTC intervention in a school environment. Third, in this study, participants perceived benefits were measured immediately after the interventions, and more information is needed on how the participants perceived benefits developed later after the intervention. Fourth, we used only one strategy/scale to measure fidelity, and the scale we used was not validated. In the future, new fidelity strategies and measures need to be developed and tested based on previous research in implementation studies. Lastly, the sample size of this study was quite small, and the participants did not differ much from each other in terms of their demographic variables. All participants were parents (mainly mothers), almost all were born in Finland and spoke Finnish as their native language, most of the parents were married/in registered partnerships/or cohabiting, had a bachelor’s or master’s degree, and were employed. It also may be that only those parents who were satisfied with the discussion participated in the study. Thus, more research is required with a larger sample size and with people with different demographic characteristics. In the future, it would also be essential to have immigrant families included.

### 4.2. Conclusion

This research showed that the LTC intervention was conducted with high fidelity, the parents’ experiences of the LTC discussion were positive, the parents felt that the atmosphere was good during the discussion, and the participants reported benefits from the intervention. The results suggest that the LTC intervention is feasible for use in a school context.

## Data availability statement

The raw data supporting the conclusions of this article will be made available by the authors, without undue reservation.

## Ethics statement

The studies involving human participants were reviewed and approved by the University of Helsinki Ethics Review Board in the Humanities and Social and Behavioral Sciences. The patients/participants provided their written informed consent to participate in this study.

## Author contributions

LA wrote the initial manuscript, conducted the data collection, and conducted the statistical analysis all with the guidance of MN, MM, and KS-A. MN, MM, and KS-A supervised the project. All authors contributed to the article and approved the submitted version.

## Funding

This study was supported by the Academy of Finland grant nos. 345117 and 336138 to KS-A, the Strategic Research Council grant no. 352509 to MN and nos. 345264 and 352660 to KS-A, the Itla Children’s Foundation grant to MN and LA. LA is also funded by grant of the Eino Jutikkala Fund of the Finnish Academy of Science and Letters.

## Conflict of interest

The authors declare that the research was conducted in the absence of any commercial or financial relationships that could be construed as a potential conflict of interest.

## Publisher’s note

All claims expressed in this article are solely those of the authors and do not necessarily represent those of their affiliated organizations, or those of the publisher, the editors and the reviewers. Any product that may be evaluated in this article, or claim that may be made by its manufacturer, is not guaranteed or endorsed by the publisher.

## Footnotes

^1^ www.mieli.fi/letstalk
